# Supplementary material for: DARTS: An Algorithm for Domain-Associated Retrotransposon Search in Genome Assemblies
Source: Genes (Basel). 2021 Dec 21;13(1):9. doi: 10.3390/genes13010009 (PMC8775202; doi:10.3390/genes13010009)
Supplement: Supplementary file 1 [file genes-13-00009-s001.zip › FigureS1.pdf]

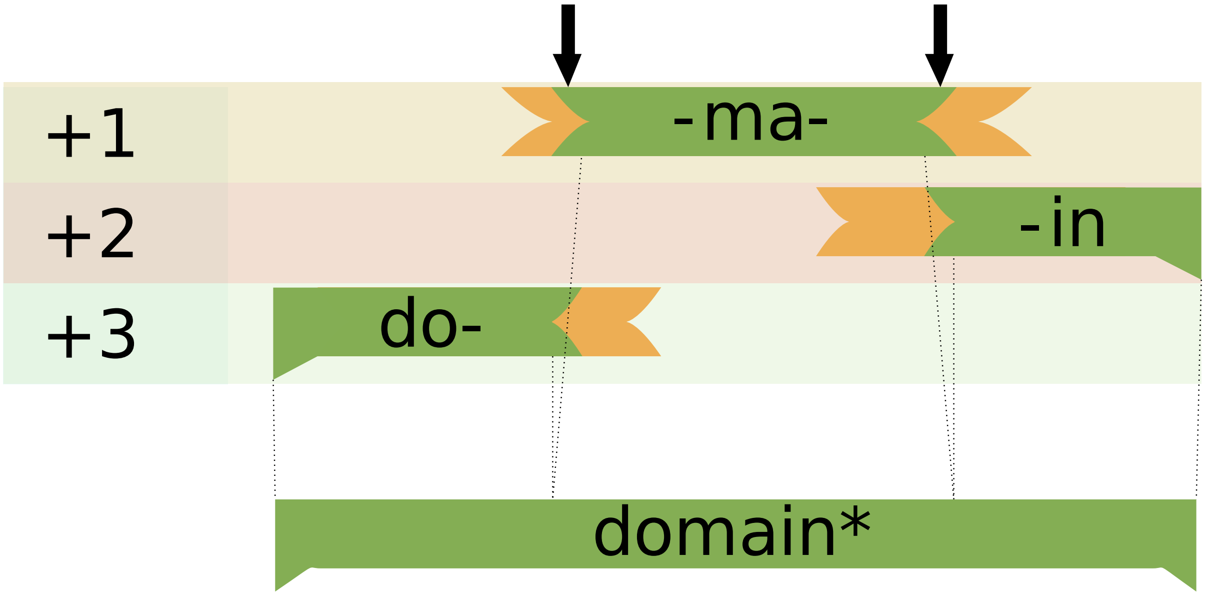

Figure S1. A principle scheme of fragmented protein domain assembly by DARTS. Translation frames are indicated as +1, +2 and +3. Black arrows indicate coordinates for merging of the fragmented domain sequences. Green regions are parts of the sequence with homology to the searched protein profile. Brown regions are non-homologous parts of the sequence. domain* - reconstructed sequence of the protein domain.
